# Expression Profile of Twelve Transcripts as a Supporting Tool for the Molecular Characterization of Canine Cutaneous Mast Cell Tumors at Diagnosis: Association with Histological Grading and Clinical Staging

**DOI:** 10.3390/genes16030340

**Published:** 2025-03-14

**Authors:** Mery Giantin, Ludovica Montanucci, Rosa Maria Lopparelli, Roberta Tolosi, Alfredo Dentini, Valeria Grieco, Damiano Stefanello, Silvia Sabattini, Laura Marconato, Marianna Pauletto, Mauro Dacasto

**Affiliations:** 1 Department of Comparative Biomedicine and Food Science, University of Padua, Viale dell’Università 16, I-35020 Legnaro, PD, Italy; rosa.lopparelli@unipd.it (R.M.L.); roberta.tolosi@unipd.it (R.T.); marianna.pauletto@unipd.it (M.P.); mauro.dacasto@unipd.it (M.D.); 2 Department of Neurology, Mc Govern Medical School, The University of Texas Health Science Center at Houston, 6431 Fannin Street, Houston, TX 44106, USA; ludovica.montanucci@uth.tmc.edu; 3 Clinica Veterinaria Tyrus, Via Aldo Bartocci 1G, I-05100 Terni, TR, Italy; alfredo.dentini@gmail.com; 4 Department of Veterinary Medicine and Animal Science, University of Milan, Via dell’Università 6, I-26900 Lodi, MI, Italy; valeria.grieco@unimi.it (V.G.); damiano.stefanello@unimi.it (D.S.); 5 Department of Veterinary Medical Sciences, Alma Mater Studiorum, University of Bologna, Via Tolara di Sopra 50, I-40064 Ozzano dell’Emilia, BO, Italy; silvia.sabattini@unibo.it (S.S.); laura.marconato@unibo.it (L.M.)

**Keywords:** canine mast cell tumor, dog, biomarker, qPCR, principal component analysis, logistic regression

## Abstract

Background/Objectives: Mast cell tumors (MCTs) are the second most common malignant neoplasms in dogs. Histopathological grading and clinical staging are the main tools for estimating biological behavior and disease extent; thus, both are essential for therapeutic decision-making and prognostication. However, the biological behavior of MCTs in dogs is variable, and it sometimes deviates from expectations. In a previous study, we identified 12 transcripts whose expression profile allowed a clear distinction between Kiupel low-grade and high-grade cutaneous MCTs (cMCTs) and was associated with prognosis. Building on these findings, this study evaluated the predictive potential of these transcripts’ expression profiles in classifying cMCTs into low-grade and high-grade. Methods: A logistic regression classifier based on the expression profiles of the identified transcripts and able to classify cMCTs as low- or high-grade was developed and subsequently tested on a novel dataset of 50 cMCTs whose expression profiles have been determined in this study through qPCR. Results: The developed logistic regression classifier reaches an accuracy of 67% and an area under the receiver operating characteristic curve (AUC) of 0.76. Interestingly, the molecular classification clearly identifies stage-IV disease (90% true positive rate). Conclusions: qPCR analysis of these biomarkers combined with the machine learning-based classifier might serve as a tool to support cMCT clinical management at diagnosis.

## 1. Introduction

Mast cell tumors (MCTs) are hematopoietic neoplasms characterized by uncontrolled proliferation and/or accumulation of neoplastic mast cells in various organs [1,2,3,4,5]. Numerous epidemiological studies conducted across various countries highlight that MCTs are one of the most frequently diagnosed malignancies, accounting for 11–17.8% of all skin neoplasia [6,7,8,9,10,11,12,13,14,15,16,17]. They appear as small, demarcated, single or multiple skin tumors that may infiltrate the surrounding tissues and metastasize to lymph nodes and internal organs like the spleen, liver, and less frequently the lungs [4,5,6,18,19].

A cytological examination of a fine needle aspirate is usually sufficient to establish the diagnosis of MCT; conversely, the biological behavior can only be determined by additional clinical and laboratory analyses [6]. Indeed, in dogs with cutaneous MCT (cMCT), the most critical prognostic indicators are histologic grade and clinical stage [6,20], which are usually integrated into the treatment decision-making. Although the biological behavior of the tumor can quite often be predicted based on clinical staging and histopathologic evaluation of the cMCT and sentinel lymph node [21], some cases deviate from the expected behavior [22]. Indeed, low-grade cMCTs with overtly metastatic lymph nodes [21], as well as non-metastatic high-grade cMCTs [23], have been reported. Thus, the molecular characterization of the tumor and the identification of potential prognostic biomarkers might support the recently improved clinical practice on cMCT [20,21,23,24,25,26].

In a previous study, we identified a molecular fingerprint useful for characterizing and prognosticating canine cMCT [27]. Through transcriptome analysis (DNA microarray) of 18 cMCT samples followed by the application of a predictive analysis, we identified a set of 13 transcripts that accurately distinguished well from poorly differentiated MCTs. These markers were also significantly associated with survival time [27]. In a subsequent study, we identified additional dysregulated genes linked to poor prognosis, specifically involved in drug metabolism and cell cycle pathways, further confirming their potential prognostic value in cMCTs [28].

In this study, we tested the predictive potential of the transcript expression profiles for classifying canine cMCTs as low-grade or high-grade. To achieve this, we developed a machine learning-based binary classifier trained on data from [27], and we tested it on a novel cohort of 50 cMCTs, which were characterized in this study through qPCR. The classifier achieved an overall accuracy of 67% and an area under the receiver operating characteristic curve (AUC) of 0.76, and its classifications significantly correlate with the WHO clinical stage. This study demonstrates the predictive potential of a small set of transcripts to serve as biomarkers for determining the molecular features (and potentially the related biological behavior) of canine cMCTs at the time of diagnosis, particularly in cases where other well-known indicators are inconclusive.

## 2. Materials and Methods

### 2.1. Gene Expression Profiling Datasets from cMCT Samples

Two gene expression profiling datasets from cMCT samples were used in this study.

Giantin2014 dataset. This dataset comprises quantitative Real-Time PCR (qPCR) data from 18 reference cMCT samples, of which 13 were classified as low-grade and 5 as high-grade, according to the Kiupel grading system [29] by three independent pathologists. Clinical stage information was not provided. This dataset is the same as that described in [27], where it was referred to as the ‘reference dataset’. In the original publication, Patnaik classification [30] and mitotic count [31] were also provided, but these data were not considered in the present study as these grading systems had either been superseded or did not contribute to or alter the current classification. In [27], we conducted a class prediction analysis using Predictive Analysis of Microarray (PAM) data and identified 13 transcripts that achieved the highest accuracy in classifying cMCTs into well-differentiated and poorly differentiated, which were hereafter referred to as low-grade and high-grade, respectively.

I-2014–2019 dataset. This dataset consists of qPCR expression data for the transcripts identified in [27] from 50 treatment-naïve new cMCT samples collected between 2014 and 2019 from academic and private veterinary clinics located in central and northern Italy. Unlike the Giantin2014 dataset, cMCT samples were histologically confirmed and graded based on the Kiupel histological grading system [29] by different diagnostic laboratories. Clinical data for each dog was available (details provided below).

### 2.2. Sample Collection and Background Information

This study, including the collection and analysis of cMCT samples, did not fall within the application areas of the Italian Legislative Decree 26/2014, which governs the protection of animals used for scientific or educational purposes; therefore, ethical approval was waived for this study. All owners signed a written informed consent.

Dogs with a previously untreated cMCT that underwent surgery between 2014 and 2019 and for which a biopsy (~30 mg of tissue core) stored at −20 °C in RNAlater solution (Applied Biosystems, Waltham, MA, USA) was available were retrospectively included.

The clinical-pathological evaluations of these cases were performed in multiple and different institutions (academic and private veterinary clinics) following the guidelines and criteria available at the time of collection. Thus, these do not completely reflect current practices or criteria updated from 2018 [20,21,24,25,26].

Background information recorded for each dog included signalment (breed, gender, age), cMCT anatomic location, clinical stage according to WHO and substage, and histological grade according to Kiupel [29]. The histological evaluation of the regional lymph node, according to Weishaar [32], was available for a subset of samples.

Information on the clinical stage was obtained by means of physical examination, hematologic and serum biochemical analyses, histological examination of the cutaneous nodule, cytological examination of regional lymph node, thoracic radiography, abdominal ultrasonography, and cytological evaluation of fine-needle aspirates from liver, spleen, and bone marrow (the last only in dogs with distant metastatic disease). For cases collected between 2014 and 2017, surgical excision and histological examination of the regional lymph node were performed only when the lymph node was enlarged or suspected to be metastatic.

### 2.3. Total RNA Isolation and KIT Mutational Analysis of the 50 cMCT Samples

Total RNA was isolated using the RNeasy Mini kit (Qiagen, Hilden, Germany) following the manufacturer’s instructions. The yield and purity of the nucleic acid were assessed by Nanodrop ND-1000 UV Spectrophotometer (Thermo Scientific, Wilmington, UK). One microgram of total RNA was reverse transcribed using the High Capacity cDNA Reverse Transcription kit (Applied Biosystems) as per the manufacturer’s instructions and stored at −20 °C until used.

The PCR amplification of *KIT* exons 8, 9, and 11 from cDNA was carried out as previously described [33]. Amplicons were visualized in a 1% agarose gel electrophoresis and sequenced on an automated sequencer (BMR Genomics, Padua, Italy).

### 2.4. Quantitative Real-Time PCR of the 50 cMCT Samples

Thirteen target transcripts, corresponding to the top differentially expressed transcripts identified using a DNA microarray approach [27], and four internal control transcripts [28] were chosen for quantitative Real-Time PCR (qPCR) amplification (Table 1). For each transcript, previously validated oligonucleotide primers and UPL probes were used [27,28]. The complete list of primers and UPL probes is reported in Table S1. The complementary DNA obtained from each specimen was amplified as previously described [27,28]. Raw data were analyzed with the LightCycler480 software release 1.5.0 (Roche) using the second derivative method. Relative quantification (RQ) values were calculated with the ∆∆Ct method [34], using the arithmetic mean of the internal control transcripts and a calibrator.

Because of the scant constitutive expression and the low amplification efficiency registered in the large majority of samples for the *NUSAP1* transcript, the raw data of this latter were censored and excluded from the subsequent analysis to avoid interpretative biases.

### 2.5. Principal Component Analysis of the Expression Profiles from the Giantin2014 Dataset

In [27], a principal component analysis (PCA) of the expression levels of the 13 transcripts in the 18 samples from the Giantin2014 dataset was conducted, demonstrating that the first three principal components (PC1, PC2, and PC3) could perfectly separate the 13 low-grade cMCTs from the 5 high-grade ones.

In this study, we repeated the PCA using expression levels from 12 of the previously identified transcripts, excluding *NUSAP1* due to technical issues (see Section 2.4). The PCA was thus applied to 18 twelve-dimensional vectors, corresponding to the expression levels of 12 transcripts (*CCNB2*, *CDC20*, *CDCA8*, *CENPP*, *FEN1*, *FOXM1*, *GSN*, *KPNA2*, *NUF2*, *PRC1*, *RAD51*, and *UBE2S*) in each of the 18 samples from the Giantin2014 dataset. The PCA was carried out through the PCA method from the *sklearn.decomposition* module [35] with default parameters, and the first 3 principal components (PC1, PC2, and PC3) were extracted.

### 2.6. Development of a Logistic Regression Classifier Based on the Expression Profiles from the Giantin2014 Dataset

Using PC1, PC2, and PC3 values from the Giantin2014 dataset as input features, we developed a logistic regression model to classify cMCTs as low-grade or high-grade, based on their expression profiles of the 12 identified transcripts. The labels with which the logistic regression model has been trained are the 18 classifications into low-grade and high-grade provided by pathologists for the Giantin2014 dataset. The binary logistic regression classifier generates a probability ranging from 0 to 1, where a prediction probability above 0.5 indicates a low-grade cMCT, and a probability below 0.5 indicates a high-grade one. The classifier was implemented using the *LogisticRegression* module from the *sklearn* package and trained on the first three PCs of samples from the Giantin2014 dataset.

### 2.7. Classification of cMCT Samples of the I-2014–2019 Dataset Through the Logistic Regression Classifier

We used the logistic regression model trained on the Giantin2014 dataset to classify samples of the I-2014–2019 dataset as low-grade or high-grade based on the expression profiles of the 12 identified transcripts. To accomplish this, we projected the expression levels of the 12 transcripts from the 50 cMCT samples of the I-2014–2019 dataset onto the PC1, PC2, and PC3 axes computed from the Giantin2014 dataset. These projections were obtained through the *transform* method of the PCA module from *sklearn.decomposition* [35]. By inputting these projections into the logistic model, we were able to classify the samples from the I-2014–2019 dataset into low-grade or high-grade. As samples from the I-2014–2019 dataset had been histologically classified into low-grade and high-grade according to [29], we used these known labels to evaluate the performances of the developed logistic regression classifier through the following indexes: accuracy, Matthews correlation coefficient, AUC, true positive and true negative rates, false positive and false negative predicted values as computed by the *confusion_matrix* module from *sklearn* [35].

### 2.8. Statistical Analysis

The Fisher exact test was applied to assess the relationship between the two predicted cMCT classes (low-grade or high-grade, as determined by the logistic regression classifier based on the expression profiles of the 12 transcripts) and WHO clinical stage. This analysis was conducted to determine whether the classification made by the logistic regression classifier aligned with a well-established prognostic indicator of the tumor. The Easy Fisher Exact Test Calculator (https://www.socscistatistics.com/tests/fisher/default2.aspx) (accessed on 16 January 2025) was used for this purpose. The level of statistical significance was set at *p* < 0.05.

## 3. Results

### 3.1. cMCT Samples of the I-2014–2019 Dataset: Caseload Description

A total of 45 dogs with 50 cMCTs were included (Table S2). The most represented breeds were Labrador retriever (*n* = 8, 17.7%) and boxer (*n* = 5, 11.1%). The remaining dogs included 10 mixed-breeds (22.2%) and 22 dogs (48.9%) from various breeds, each represented once or twice. The median age was 8 years (range, 2–15 years). There were 31 females (16 spayed) and 14 males (2 neutered). Caseload data (i.e., dog breed, age, and gender) are representative of the Italian population of dogs affected by skin tumors [14,17].

Regarding clinical staging, 9 dogs (18.0%) had WHO stage I disease, 19 (38.0%) had stage II, 8 (16.0%) had stage III, and 8 (16.0%) had stage IV. Thirty-seven (82.2%) dogs were asymptomatic (substage a), while seven (15.6%) had substage b disease. The clinical stage was not available for one case.

Seven dogs had multiple cMCTs, and biopsies were obtained from two different nodules in five of these cases. Tumor locations included limbs (*n* = 19, 38.0%), head and neck (*n* = 14, 28.0%), vulva (*n* = 4, 8.0%), trunk (*n* = 2, 4.0%), perineal region (*n* = 2, 4.0%), foreskin (*n* = 2, 4.0%), axilla (*n* = 1, 2.0%), inguinal region (*n* = 1, 2.0%) and mammary gland (*n* = 1, 2.0%). The tumor location was not reported for four cases (7.8%).

Histologically, based on the Kiupel grading, 32 cMCTs (64.0%) were classified as low-grade and 11 (22.0%) as high-grade, with the grade unavailable for 7 samples. Data regarding the histological evaluation of the regional lymph node [32] were provided for a subgroup of 21 cMCT samples obtained from 18 dogs. Lymph node evaluation showed 8 non-metastatic (HN0-1) cases, which included 6 stage I disease, and 2 stage III (7 low-grade and 1 high-grade) and 10 metastatic (HN2-3) cases, including 7 stage II, 1 stage III, and 2 stage IV (8 low-grade and 2 high-grade).

Missense single nucleotide polymorphisms (SNPs) and insertion/deletions (INDELs) on *KIT* cDNA were observed in 8 out of 50 specimens (16.0%). Specifically, the ITD417-420 was identified in one low-grade cMCT, while the INDELs DEL546-552, p.(Lys557Asn) + DEL558-559, ITD572-586, ITD573-585, p.(Tyr573Asp) + ITD574-585, ITD575-589, and ITD578-591 were equally detected in three Kiupel low-grade and three Kiupel high-grade, and in one ungraded cMCT (one INDEL per case).

### 3.2. Principal Component Analysis of the Gene Expression Data from the Giantin2014 Dataset

**Figure 1:**
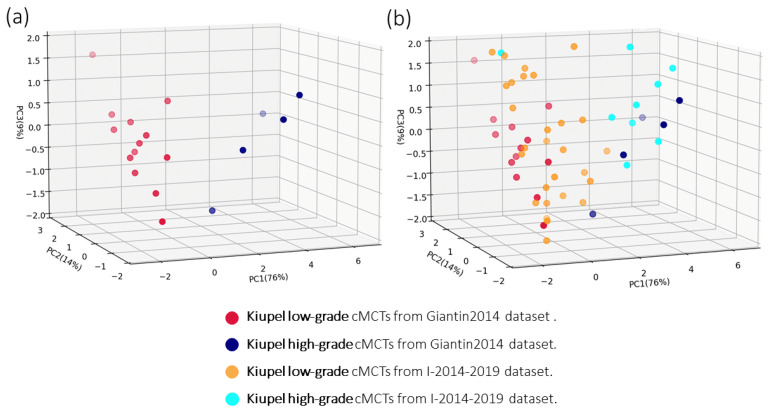
Principal Components Analysis of Expression Profiles from 12 Transcripts in cMCT Samples. (**a**) PCA of the Giantin2014 dataset, illustrating 13 low-grade (red dots) and 5 high-grade (blue dots) cMCTs, using the expression profile of the 12 target transcripts identified in [27] (listed in Table 1). (**b**) PCA of the Giantin2014 dataset and projection of 50 new cMCT samples from the I-2014–2019 dataset. Red and blue dots represent histologically classified low-grade and high-grade samples from the Giantin2014 dataset, respectively. Orange and cyan dots represent histologically classified low-grade and high-grade cMCT samples from the I-2014–2019 dataset, respectively. The transparency of each dot provides a visual cue for its relative position in the third dimension.

The mRNA expression levels for the 13 identified transcripts in the 18 reference cMCTs of the Giantin2014 dataset are provided in [27]. In this study, we repeated the PCA using the mRNA expression levels of 12 transcripts, excluding the *NUSAP1* transcript due to technical issues (see Methods). A visualization of the cMCTs from the Giantin2104 dataset onto the obtained PC1, PC2, and PC3 is shown in Figure 1a. The first three principal components account for 76%, 14%, and 9% of the total variance, respectively, and collectively explain 99% of the variance in the expression levels of the 12 transcripts. Figure 1a shows that the first three principal components of the expression levels of the 12 target transcripts (even after removing the expression levels of the *NUSAP1*) are sufficient to clearly separate the 13 low-grade (red dots) from the 5 high-grade (blue dots) cMCT samples of the Giantin2014 dataset.

### 3.3. Test of the Logistic Regression Classifier on cMCTs from the I-2014–2019 Dataset

The mRNA expression levels of the 12 target transcripts for the 50 cMCTs of the I-2014–2019 dataset were measured using qPCR, with the resulting relative quantification (RQ) values presented in Supplementary Table S3. The projections of these expression profiles onto the previously identified principal components (PC1, PC2, and PC3) are illustrated in Figure 1b, with orange and cyan dots representing low-grade and high-grade cMCTs from the I-2014–2019 dataset, respectively.

We then used these PCA projections and the logistic regression classifier to classify the cMCT samples of the I-2014–2019 dataset into low-grade and high-grade. In total, 26 cMCT samples from 22 dogs were classified as low-grade, and 24 specimens from 23 dogs were classified as high-grade. The developed logistic classifier reached an accuracy of 67% and an AUC of 0.76 and its performance is reported in Table 2. Table 2 shows that the accuracy is unevenly distributed between the two classes, with the true positive rate (TPR) for the low-grade class being higher than the true negative rate (TNR) for the high-grade class.

### 3.4. Association Between the Expression Profile-Based Classification and the WHO Clinical Stage in 50 cMCTs

To explore the potential association (contingency) between the linear regression classification based on expression profiles and the clinical stage, we applied a Fisher’s exact test. The two classification groups (low-grade and high-grade) were assessed against the WHO clinical stage. The results for all 50 cMCT samples are shown in Table 3. The expression profile-based classification was found to be significantly associated with the WHO clinical stage (*p* < 0.05). Indeed, the cMCT group predicted as high-grade contained the majority of stage IV cases (7 out of 8; 87.5%).

## 4. Discussion

Cutaneous MCTs exhibit significant variability in their biological behavior and prognosis, presenting challenges in diagnosis and management. Veterinary oncologists typically depend on histological grading and clinical staging to assess prognosis and determine the most appropriate treatment strategy [6,7,18,20]. Despite advances in the understanding of cMCTs, certain cases remain particularly challenging to manage [20,22,23]. This underscores the need for innovative indicators to further support clinical decision-making. Recently, specific biologic variables (i.e., primary tumor diameter, ulceration, regional/sentinel lymph node status, and distant metastasis) have been successfully integrated into a proposed new clinical staging system for canine MCT [20].

In this study, we investigated whether the expression profiles of 12 transcripts could serve as a straightforward molecular tool to complement histological grading and clinical staging. This approach aims to assist clinicians in characterizing canine cMCTs at diagnosis and potentially predicting their biological behavior.

Molecular biomarkers are gaining traction in veterinary medicine and are anticipated to play a central role in precision care for tumor-bearing dogs in the future [36]. As a matter of fact, the recent advances in the comprehension of the canine genome provided new opportunities to enhance the knowledge on the molecular basis (both pathogenesis and progression) of dog cancers [27,28,37,38,39,40,41,42,43,44,45,46,47,48,49,50,51,52,53,54,55,56]. Gene expression analysis, further than DNA methylation, copy number aberration, and mutational profiling, allowed the identification of biomarkers reflecting particular biological properties of the neoplasm (e.g., proliferative capacity and metastatic potential) that are useful to segregate tumors/clinical cases into subgroups based on likely disease-course [57]. Focusing specifically on MCT, a recent paper on the genome-wide gene expression characterization of 15 MCT samples by RNA-seq identified two distinct tumor subtypes (i.e., high-risk and low-risk), differing for 71 differentially expressed transcripts, that were associated with histological grade, survival time, Ki67 index, and occurrence of MCT-related death [54].

In the present work, we took advantage of a preliminary deep molecular characterization of cMCTs [27,28] and evaluated the potential use of a reduced gene set, transferable to a diagnostic platform, to rapidly and accurately distinguish at diagnosis the two molecular subtypes of low-grade and high-grade cMCTs. Frantz and colleagues previously applied a similar approach, using gene expression analysis of a limited set of transcripts to classify canine lymphoma samples into three subgroups with distinct molecular characteristics [42]. For the current study, we focused exclusively on transcripts identified through rigorous class prediction analysis, where reference samples were selected solely based on histological features [27]. We excluded *SLC38A8* and *UGT2A1*, the main transcripts identified in our outcome-based study [28], due to their low expression in a large number of cMCTs examined here and to avoid biases related to the lack of standardization in staging and treatment procedures at the time of sample collection and analysis [28].

The 12 target genes considered in this study, *CCNB2*, *CDC20*, *CDCA8*, *CENPP*, *FEN1*, *FOXM1*, *GSN*, *KPNA2*, *NUF2*, *PRC1*, *RAD51*, and *UBE2S*, are mainly involved in cell cycle, DNA replication, p53 signaling pathway, nucleotide excision repair, and pyrimidine metabolism [27]. Except for the oncosuppressor *GSN* [58] that showed an opposite behavior, all the other transcripts were overexpressed in high-grade cMCT specimens vs. low-grade ones. This indicates a higher proliferation rate, malignant transformation, and an increased response to DNA damage events in more aggressive cMCTs [27]. Interestingly, and in accordance with these results, copy number gains of *FOXM1* and *RAD51* genes have been previously observed in MCT-affected dogs, showing a poor outcome [59]. All the remaining genes, mostly involved in the cell cycle and playing a pivotal role in tumor progression, were previously shown to be overexpressed in a variety of human cancers [60,61,62,63,64,65,66,67,68,69,70,71,72,73,74,75,76,77,78,79,80,81]. Worth mentioning, other authors, using another analytical approach (i.e., RNA-seq), in high-risk MCTs identified differentially expressed transcripts belonging to biological processes related to the positive regulation of cell proliferation (e.g., cell cycle process, mitotic cell cycle process, regulation of chromosome segregation, and regulation of cell cycle) as in [27], in addition to extracellular matrix-related terms (functions of cancer-associated fibroblasts) [54].

In addition to advanced molecular tools, artificial intelligence is also transforming cancer research and precision medicine [82]. In oncology research, the applications of artificial intelligence range from the detection and classification of neoplasia to the characterization of tumors and their microenvironment, and the prediction of treatment outcomes for patients [83]. As an example, in veterinary oncology, a machine-learning-based algorithm for the automated diagnosis of seven of the most common canine skin tumors (MCT comprised) was recently described [84]. Focusing specifically on transcriptomic data, Cheng and collaborators implemented a machine-learning application from RNA-seq data of canine hemangiosarcoma tumor samples and nonmalignant tissues for diagnostic purposes [56].

In this intriguing scientific context, the novelty of our proof-of-concept study is the application of a linear machine learning method on the PCA components derived from qPCR results. To build this model, we used the gene expression data of the 18 reference cMCT samples (13 low-grade and 5 high-grade, named here Giantin2014 dataset), previously classified exclusively using histological criteria. This analysis represents the first attempt to classify cMCT specimens in subgroups based on a small number of molecular features (*n* = 12 transcripts).

Despite the small size of the training dataset, which limits the generalizability of the findings and may affect the robustness of the machine learning model, our logistic regression classifier achieved a notably high performance, with an AUC of 0.76, which aligns with previously published findings [29,85]. This result underscores the strong predictive potential of the expression profile of the 12 identified transcripts, supporting their possible role as biomarkers for the histological grading of canine cMCTs. Between the two classes (i.e., Kiupel high-grade and Kiupel low-grade), the highest classification performance is achieved for the class of the Kiupel high-grade, as the vast majority (90%) of cMCT samples are correctly classified. Conversely, a consistent percentage of low-grade tumors were classified by the logistic regression classifier as high-grade. This last result, on one side, confirms the well-known extreme heterogeneity of cMCTs [6,7,18,19] and indicates the potential limits of the classifier that could be smoothed by increasing the number of reference samples; on the other side, it might highlight the intrinsic molecular features of a small percentage of low-grade tumors that possess a higher aggressiveness or metastatic potential, as previously described by [29]. In line with this hypothesis, Stefanello and collaborators [85] reported that, in a cohort of 295 dogs with Kiupel low-grade tumors, 44 (14.9%) had metastatic disease. Thus, this set of 12 markers may highlight biological processes that diverge from canonical clinical-pathological observations, including nodal metastasis—absent from this series but typically highly indicative of prognosis [20,21,24,25].

Despite the Giantin2014 dataset derived from the qPCR analysis of 18 reference cMCTs classified using only histological parameters, in this study, we additionally attempted to evaluate any potential association of the molecular classification with the clinical stage. Indeed, a statistically significant association between the two classification criteria was obtained. Specifically, the linear machine learning algorithm was able to classify seven out of eight stage IV cMCTs (87.5%) as high-grade. This was quite expected since stage IV disease is usually associated with high histological grade, better than clinical signs of aggressive behavior and poor prognosis [4]. Out of the 36 cMCTs classified as stage I-III, 16 (44.4%) were also labeled as high-grade. This result may partly reflect the limitations of histological grading but is likely influenced by the clinical staging criteria used during sampling, particularly between 2014 and 2017. At that time, staging relied on the WHO system, which focuses on macroscopic tumor features, the presence of multiple tumors, nodal involvement, and distant metastasis [86]. Notably, nodal status, a critical factor for prognosis and treatment decisions [20,22,24,26,32], was unavailable for half of the cases. Acknowledging these limitations, we re-evaluated the association between the expression profile-based classification and the clinical stage, focusing exclusively on cMCT cases with histological lymph node evaluation. However, the results were inconclusive due to the small sample size (*n* = 18), the limited representation of Kiupel high-grade tumors (*n* = 3), and the underrepresentation of stage III-IV cMCTs (*n* = 3 and *n* = 2, respectively). Future reanalysis with larger multi-center datasets and more standardized, contemporary classification protocols will be essential to further clarify the predictive utility of these 12 transcripts as biomarkers for histological and/or clinical staging.

In perspective, to evaluate the potential additional prognostic value of the molecular tool, integration with follow-up data is crucial. In this study, survival time and time to progression data were collected, but they were deemed unreliable due to several factors: (a) the multi-institutional nature of the study; (b) the lack of standardization in data collection, clinical staging and therapy; and (c) the time frame of sample collection (2014–2019) during which clinical staging protocols and standard care practices for cMCT underwent significant advances [20,21,24,25,26]. Thus, the molecular classification presented in this study should currently be regarded as a complementary diagnostic tool to support established histological and clinical evaluations. It may be particularly valuable in cases where histopathological grading or clinical staging yields inconclusive results or when recommended clinical staging cannot be performed due to owner non-compliance or financial constraints.

*KIT*-activating mutations are among the prognostic factors considered in canine MCT (reviewed in [6]). While ITDs in exon 8 have been more frequently identified in less aggressive MCTs [87,88], gain-of-function mutations (both missense SNVs and INDELs) have been associated with a higher risk of recurrence and metastasis, MCT-related mortality and a shorter survival time [22,89,90,91]. In the present study, *KIT*-activating mutations were identified in 17.7% of cMCT cases, and they were randomly distributed within the samples classified by the machine learning algorithm as low-grade and high-grade. This result was probably influenced by the low number of tested samples, or it could be related to the occurrence of *KIT* mutations independently from the biological processes described by the 12 selected transcripts. Hence, a higher number of samples should be analyzed and a more detailed analysis of the potential interactions of *KIT*-activating mutations with the 12 transcripts might be explored in perspective to provide additional insights into cMCT biology.

Finally, the classifier’s overall performance might have been affected by the omission of the *NUSAP1* transcript, which was excluded due to technical constraints (specifically, low amplification efficiency). Furthermore, while qPCR is a cost-effective method for gene expression analysis, it may not provide a comprehensive view of the transcriptome as more sophisticated techniques such as RNA-seq.

In conclusion, this study introduces a simple, rapid, and cost-effective qPCR-based tool for characterizing the molecular profile of canine cMCTs. Using a machine learning approach, the classification into low-grade and high-grade cMCTs showed promising results, significantly correlating with Kiupel histological grading and clinical staging. After further validation with a larger dataset and comprehensive clinical and follow-up data, this tool could aid veterinary oncologists in predicting cMCT biological behavior, especially in cases where histological grading or clinical staging is inconclusive or incomplete. Given the limitations of this study—such as small sample size, lack of comprehensive follow-up data, and incomplete clinical staging—future research addressing these weaknesses will be crucial for validating and refining this tool for use in veterinary oncology.

## Figures and Tables

**Table 1:** List of the 13 target and 4 internal control transcripts considered in the present study.

Transcript	Description	Ensembl Genome Browser Transcript ID
Target transcripts		
*CCNB2*	Cyclin B2	ENSCAFT00000026290
*CDC20*	Cell division cycle 20	ENSCAFT00000008495
*CDCA8*	Cell division cycle associated 8	ENSCAFT00000005257
*CENPP*	Centromere protein P	ENSCAFT00000003607
*FEN1*	Flap structure specific endonuclease 1	ENSCAFT00000049322
*FOXM1*	Forkhead box M1	ENSCAFT00000024793
*GSN*	Gelsolin	ENSCAFT00000005907
*KPNA2*	Karyopherin α 2	ENSCAFT00000018435
*NUF2*	NDC80 kinetochore complex component, homolog	ENSCAFT00000021052
*NUSAP1*	Nucleolar and spindle-associated protein 1	ENSCAFT00000015151
*PRC1*	Protein regulator of cytokinesis 1	ENSCAFT00000019302
*RAD51*	DNA repair protein RAD51 homolog 1	ENSCAFT00000014658
*UBE2S*	Ubiquitin-conjugating enzyme E2S	ENSCAFT00000045087
Internal control transcripts		
*CCZ1*	CCZ1 homolog, vacuolar protein trafficking, and biogenesis associated	ENSCAFT00845017131
*GUSB*	Glucuronidase β	ENSCAFT00000062136
*RPL8*	Ribosomal protein L8	ENSCAFT00000002627
*RPS5*	Ribosomal protein S5	ENSCAFT00000109444

**Table 2:** Performances of the logistic regression classifier on the test dataset I-2014–2019.

Accuracy	AUC	MCC	TNR	NPV	TPR	PPV
0.674	0.761	0.428	0.606	0.952	0.900	0.409

The performances of the logistic regression classifier were computed on the 43 (out of 50) cMCTs for which Kiupel histological classification was provided (see Section 3.1). Accuracy is the total number of corrected classifications; AUC is the area under the receiver operating characteristic curve; MCC is the Matthews correlation coefficient; TPR and TNR are the true positive and negative rates, respectively; PPV and NPV are the positive and negative predicted values, respectively.

**Table 3:** Association of the expression profile-based classification with WHO clinical stage.

Clinical Stages	Samples	cMCTs Predicted as Low-Grade	cMCTs Predicted as High-Grade	Fisher Exact Test (*p*)
I-II-III	36	20	16	0.0481 *
IV	8	1	7	
NP	1	1	0	

NP: not provided. *: *p* < 0.05.

## Data Availability

The original contributions presented in this study are included in the article/Supplementary Materials. Further inquiries can be directed to the corresponding author.

## Supplementary Materials

The following supporting information can be downloaded at: https://www.mdpi.com/article/10.3390/genes16030340/s1, Table S1: Oligonucleotide primers and UPL probes used in the present study; Table S2: Case load: signalment, WHO clinical stage, Kiupel histological grade, nodal status and *KIT* mutational status of the cutaneous mast cell tumors considered in the present study (I-2014–2019 dataset); Table S3: Relative quantification (RQ) values of the target genes (*n* = 12) in 50 cutaneous mast cell tumor samples (I-2014–2019 dataset).
