# Supplementary material for: Expression Profile of Twelve Transcripts as a Supporting Tool for the Molecular Characterization of Canine Cutaneous Mast Cell Tumors at Diagnosis: Association with Histological Grading and Clinical Staging
Source: Genes (Basel). 2025 Mar 14;16(3):340. doi: 10.3390/genes16030340 (PMC11942052; doi:10.3390/genes16030340)
Supplement: Supplementary file 1 [file genes-16-00340-s001.zip › genes-3488633-supplementary.pdf]

Article

Expression Profile of Twelve Transcripts as a Supporting Tool for the Molecular Characterization of Canine Cutaneous Mast Cell Tumors at Diagnosis: Association with Histological Grading and Clinical Staging

Mery Giantin [1,*,†], Ludovica Montanucci [2,†], Rosa Maria Lopparelli [1], Roberta Tolosi [1], Alfredo Dentini [3], Valeria Grieco [4], Damiano Stefanello [4], Silvia Sabattini [5], Laura Marconato [5], Marianna Pauletto [1] and Mauro Dacasto [1]

1 Department of Comparative Biomedicine and Food Science, University of Padua, Viale dell'Università 16, I-35020 Legnaro, PD, Italy; rosa.lopparelli@unipd.it (R.M.L.); roberta.tolosi@unipd.it (R.T.); marianna.pauletto@unipd.it (M.P.); mauro.dacasto@unipd.it (M.D.)

2 Department of Neurology, Mc Govern Medical School, The University of Texas Health Science Center at Houston, 6431 Fannin Street, Houston, TX 44106, USA; ludovica.montanucci@uth.tmc.edu

3 Clinica Veterinaria Tyrus, Via Aldo Bartocci 1G, I-05100 Terni, TR, Italy; alfredo.dentini@gmail.com

4 Department of Veterinary Medicine and Animal Science, University of Milan, Via dell'Università 6, I-26900 Lodi, MI, Italy; valeria.grieco@unimi.it (V.G.); damiano.stefanello@unimi.it (D.S.)

5 Department of Veterinary Medical Sciences, Alma Mater Studiorum, University of Bologna, Via Tolara di Sopra 50, I-40064 Ozzano dell'Emilia, BO, Italy; silvia.sabattini@unibo.it (S.S.); laura.marconato@unibo.it (L.M.)

* Correspondence: mery.giantin@unipd.it; Tel.: +39-049-8272946

† These authors contributed equally to this work.

Supplementary files

Table S1. Oligonucleotide primers and UPL probes used in the present study.

Gene	Primer sequence (5’- 3’)	UPL probe (nr)	Amplicon size (bp)	Reference
CCNB2	F: CCAGTACAGATGGAAATGTTGG<br>R: AGGTTCTCTTCCTTCATGGAGAT	81	77	Giantin et al. 2014
CDC20	F: GTGCCGTGGATGCTCAAT<br>R: CCAGAGATGAGCTCCTTGTAGTG	54	70	Giantin et al. 2014
CDCA8	F: GGTTTGACTCAAGGGTCTTCA<br>R: GAGATGTTGTAGATCCGCTCTCT	3	73	Giantin et al. 2014
CENPP	F: TTCCGAAGCTGGATCTTCTC<br>R: AGTCTCCAGGATGTGGTGCT	1	74	Giantin et al. 2014
FEN1	F: AGGAGCAATTTGTAGATCTGTCG<br>R: CCCGAATGCTCTCACAGTAGT	3	60	Giantin et al. 2014
FOXM1	F: CGAGGATCACTTCCCCTATTT<br>R: GAAAGGTTGTGGCGGATG	11	72	Giantin et al. 2014
GSN1	F: CCTGGGACAGCTTCAACAAC<br>R: CCGCACCACTGGTAGATGT	61	70	Giantin et al. 2014
KPNA2	F: TGTCAAAGGCATAAATAGCAACA<br>R: AGCAGTTTCCTAGCAGCTTGA	1	69	Giantin et al. 2014
NUF2	F: ATGAAAGACACAGTCCAGAAACTT<br>R: GCAAGCAATCAACGGAGTCT	137	89	Giantin et al. 2014
NUSAP1	F: GCCTACCTTAAAGACGAAGCAA<br>R: CATCTGCATCTCAGTCTCATCC	88	93	Giantin et al. 2014
PRC1	F: CAACGAGCCAAGCTTCAAA<br>R: ATGTTTCAATCCGTGCCTTC	144	67	Giantin et al. 2014
RAD51	F: GGCCATGTACATTGACACTGA<br>R: CACTGCCAGAGAGGCCATA	102	83	Giantin et al. 2014
UBE2S	F: GCATGTCCTGCTGACCATC<br>R: CTCATTGAGGGCCGACTC	40	64	Giantin et al. 2014
CCZ1	F: TGAAGCACTGCATTTAATTGTTTAT<br>R: CTTCGGCAAAAATCCAATGT	136	96	Giantin et al. 2016
GUSB	F: CGACATCACCGTCACCAC<br>R: CACTGCCCTGGACAAAAATC	26	75	Giantin et al. 2016
RPL8	F: GGACGGAGCTGTTCATCG<br>R: GCACATTGCCTATGTTGAGC	137	90	Giantin et al. 2016
RPS5	F: CCGGAACATCAAGACTATTGC<br>R: GAATTGGAAGAGCCCTTGG	136	72	Giantin et al. 2016

Table S2. Caseload: signalment, WHO clinical stage, Kiupel histological grade, nodal status and *KIT* mutational status of the cutaneous mast cell tumors considered in the present study (I-2014-2019 dataset).

CASE Nr.	BREED	GENDER	AGE (yy)	ANATOMIC LOCATION	WHO CLINICAL STAGE	WHO CLINICAL SUBSTAGE	KIUPEL HISTOLOGICAL GRADE	NODAL STATUS (Weishaar et al., 2014)	KIT MUTATIONAL STATUS
**1**	Dogo Argentino	MC	7	shoulder	IV	b	low-grade	HN3	c.1731C>T p.(Tyr577=)
**2**	Boxer	F	6	planum nasale	II	a	low-grade	HN3	WT
**3**	French Bulldog	F	4	ear	I	a	low-grade	HN0	WT
**4**	Pug	FS	7	vulva	I	a	low-grade	HN0	WT
**5**	mixed-breed	F	13	-	IV	b	high-grade	-	WT
**6**	Pug	M	6	multicentric MCT – thigh	III	a	-	-	WT
**7**	Labrador retriever	FS	8	lip	II	a	high-grade	HN3	ITD572-586
**8**	mixed-breed	FS	11	hip	I	a	low-grade	-	WT
**9**	Dogo argentino	FS	9	multicentric MCT – vulva	III	a	-	-	WT
**10A-B**	English Setter	F	7	multicentric MCT – hip and thigh	III	b	low-grade	HN2	WT
**11**	English Setter	M	7	shoulder	I	a	low-grade	HN0	WT
**12**	mixed-breed	F	14	axilla	II	b	high-grade	-	WT
**13**	Boxer	FS	10	vulva	II	a	low-grade	HN2	WT
**14**	English Setter	FS	9	lip	II	a	high-grade	-	WT
**15**	Labrador	F	13	shoulder	II	a	-	-	WT
**16**	Australian terrier	F	11	mammary gland	II	a	low-grade	-	p.(Lys557Asn) + DEL558-559

**17**	Labrador retriever	FS	8	abdomen	II	a	low-grade	HN3	WT
**18**	mixed-breed	F	13	-	-	-	-	-	c.1731C>T p.(Tyr577=)
**19**	Labrador retriever	FS	7	shoulder	I	a	high-grade	-	WT
**20**	Labrador retriever	FS	11	hock	II	a	low-grade	-	WT
**21**	Labrador retriever	M	6	facial muscle	IV	a	low-grade	-	ITD578-591
**22**	mixed-breed	FS	2	ear base	II	a	high-grade	-	p.(Tyr573Asp) + ITD574-585
**23**	Labrador retriever	M	8	ear	I	a	low-grade	-	WT
**24**	Beagle	M	12	knee	II	a	low-grade	-	WT
**25**	Bullmastiff	MC	6	thigh	II	a	low-grade	HN2	WT
**26**	Weimaraner	F	7	stifle	I	a	low-grade	HN0	WT
**27**	Boxer	M	11	eyelid	II	a	low-grade	-	WT
**28**	Bernese mountain dog	F	11	-	IV	b	-	-	DEL546-552
**29**	Amstaff	FS	5	-	II	a	low-grade	-	WT
**30**	Boxer	F	8	ear	II	a	low-grade	-	WT
**31**	Pinscher	F	9	thigh	IV	a	high-grade	-	WT
**32**	Shar pei	F	10	neck	III	a	low-grade	HN2	WT
**33**	mixed-breed	M	14	foreskin	IV	b	high-grade	-	WT
**34**	Labrador retriever	F	8	thorax	IV	a	high-grade	-	WT

**35A-B**	mixed-breed	M	10	multicentric MCT – peripeneal region 1 and 2	III	a	-	-	WT
**36**	Maltese	FS	15	thigh	II	a	low-grade	-	WT
**37A-B**	Schnauzer	FS	8	multicentric MCT – left and right ear	III	a	low-grade	HN0	WT
**38A**	Boxer	M	7	multicentric MCT – thigh	III	a	low-grade	-	ITD417-421
**38B**	Boxer	M	7	multicentric MCT – foreskin	III	a	low-grade	-	WT
**39**	Alaskan Malamute	F	4	thigh	II	a	low-grade	HN3	WT
**40A**	French Bulldog	M	8	multicentric MCT – thigh	III	a	low-grade	HN1	c.1731C>T p.(Tyr577=) ITD575-589
**40B**	French Bulldog	M	8	multicentric MCT – hock	III	a	low-grade	HN1	c.1731C>T p.(Tyr577=)
**41**	mixed-breed	FS	11	inguinal region	IV	b	high-grade	HN3	ITD573-585
**42**	mixed-breed	M	14	eyelid	I	a	high-grade	HN0	WT
**43**	Bleu de Guascoine	FS	11	emitorax	II	a	low-grade	-	WT
**44**	mixed-breed	F	6	vulva	II	a	low-grade	-	WT
**45**	Golden retriever	FS	3	ear	I	a	low-grade	HN0	WT

M: male; MC: male, castrated; F: female; FS: female, spayed; DEL: deletion; ITD: internal tandem duplication; SNP: single nucleotide polymorphism; WT: wild type genotype.

Table S3. Relative quantification values (RQ) of the target genes (n = 12) in 50 cutaneous mast cell tumor samples (I-2014-2019).

Sample ID	*CCNB2*	*CDC20*	*CDCA8*	*CENPP*	*FEN1*	*FOXM1*	*GSN*	*KPNA2*	*NUF2*	*PRC1*	*RAD51*	*UBE2S*
**1**	0.223	0.511	0.467	0.576	0.543	1.113	1.149	1.361	1.202	0.880	0.337	0.693
**2**	0.127	0.393	0.177	0.594	1.759	7.945	2.250	16.450	0.527	0.741	0.518	1.102
**3**	0.229	1.095	0.520	7.523	0.940	2.405	0.443	1.434	2.587	0.936	1.065	0.911
**4**	0.316	2.223	0.665	8.014	0.424	1.033	3.026	1.255	0.188	0.342	0.353	1.392
**5**	7.938	4.066	3.874	0.180	1.056	0.317	0.795	0.145	3.503	3.314	4.450	2.621
**6**	3.640	1.801	1.669	0.132	0.444	0.241	1.494	0.163	0.923	1.595	1.788	1.400
**7**	4.980	1.995	2.364	0.173	0.495	0.202	0.262	0.179	0.940	1.761	2.841	2.580
**8**	0.735	0.517	0.800	0.929	0.362	0.280	2.600	0.220	0.332	0.414	0.381	0.330
**9**	2.523	0.973	1.613	0.322	0.886	0.475	5.657	0.497	0.534	1.214	1.414	0.448
**10A**	0.186	0.579	0.919	1.100	0.696	0.328	3.476	1.250	0.334	0.436	0.392	0.822
**10B**	0.251	0.907	0.515	1.458	0.613	0.564	3.942	1.136	0.404	0.768	0.312	0.632
**11**	0.515	1.271	0.631	1.022	0.772	0.454	3.827	0.957	0.527	0.700	0.421	1.207
**12**	0.389	0.842	0.802	1.388	0.665	0.691	4.635	1.233	0.805	0.526	0.811	1.005
**13**	0.145	0.606	3.549	0.081	1.856	5.990	0.462	10.574	0.912	0.469	0.466	0.875
**14**	1.755	2.508	3.854	0.130	0.772	0.149	1.386	0.110	1.036	1.302	2.030	2.607
**15**	2.047	0.132	0.441	0.363	0.419	0.182	2.926	0.290	0.130	0.190	0.524	0.495
**16**	5.464	0.330	1.185	0.463	0.306	0.075	1.580	0.180	0.112	0.159	0.449	0.411
**17**	0.215	1.557	2.591	0.514	0.632	1.269	0.697	2.758	1.013	0.942	0.755	1.692
**18**	4.392	0.155	0.409	0.151	0.354	0.432	2.211	0.467	0.150	0.183	0.322	0.315
**19**	1.175	3.765	1.876	1.508	0.863	1.427	1.221	0.968	0.935	0.456	1.915	1.627
**20**	0.092	0.043	0.829	1.209	0.358	0.402	2.460	1.256	0.254	0.175	0.301	2.240
**21**	0.565	3.500	10.429	239.271	5.531	19.260	0.370	40.294	2.246	0.587	0.866	2.215
**22**	2.536	3.255	1.286	1.163	2.089	5.550	0.357	5.493	5.806	3.323	2.607	2.589
**23**	0.846	1.060	0.855	2.538	0.687	0.952	1.629	1.964	1.010	0.816	0.806	1.877
**24**	1.562	6.492	4.623	3.114	0.876	1.832	1.438	1.408	1.256	1.686	1.128	3.008
**25**	0.818	0.435	1.315	0.868	0.467	0.141	1.469	0.180	0.337	0.753	1.803	0.339
**26**	0.064	0.304	0.201	0.190	0.303	0.783	0.657	0.525	0.431	0.135	0.158	0.146
**27**	0.091	0.299	0.805	0.827	0.189	1.430	0.411	0.435	5.642	0.714	1.554	1.362

**28**	6.158	3.393	10.722	0.134	0.721	0.372	0.309	0.258	2.358	4.091	5.417	2.916
**29**	8.724	7.808	5.370	0.180	0.519	0.315	0.473	0.154	2.403	2.576	3.010	2.229
**30**	0.088	0.718	0.504	0.746	0.652	0.689	4.035	0.931	0.135	0.163	0.319	1.350
**31**	1.005	4.206	2.031	3.166	0.731	1.350	2.231	1.171	1.191	0.527	0.564	1.844
**32**	0.364	1.140	1.075	5.076	0.308	0.444	3.455	0.473	0.252	0.109	0.340	0.706
**33**	8.070	12.974	8.649	0.736	3.938	3.897	0.118	2.424	1.738	2.756	3.047	5.292
**34**	1.679	4.049	2.843	3.476	1.482	2.589	0.925	3.739	1.714	1.204	1.187	2.301
**35A**	0.638	0.465	0.467	0.059	0.233	0.060	1.755	0.063	0.111	0.184	0.499	0.964
**35B**	2.587	0.833	1.062	0.260	0.282	0.212	1.481	0.200	0.130	0.877	1.076	0.608
**36**	0.711	2.259	1.313	3.100	1.104	3.830	0.529	4.916	3.649	1.208	0.617	2.046
**37A**	0.273	0.790	1.003	4.773	0.815	0.572	2.242	1.185	0.188	0.088	0.603	0.997
**37B**	0.250	0.703	0.656	1.290	0.550	0.317	4.848	1.059	0.293	0.343	0.158	0.928
**38A**	0.067	0.146	0.133	1.072	0.490	0.719	1.385	1.505	0.415	0.812	0.213	0.838
**38B**	0.311	0.355	4.212	33.119	4.712	14.038	0.379	12.391	2.623	3.390	0.322	0.523
**39**	0.422	0.744	0.112	2.460	0.650	2.352	0.765	1.813	2.098	1.323	0.469	1.201
**40A**	0.757	1.714	1.054	0.754	0.567	1.187	2.317	0.951	0.866	0.665	0.958	1.163
**40B**	0.437	1.040	1.435	1.406	1.420	2.669	3.962	2.499	0.434	0.553	0.559	1.254
**41**	5.192	2.678	8.792	0.125	0.300	0.172	0.232	0.088	1.084	0.200	2.307	0.207
**42**	1.161	1.313	5.364	22.009	3.074	11.917	1.213	7.674	25.614	6.908	1.197	3.681
**43**	0.367	0.834	1.472	1.431	0.783	1.074	4.539	1.417	0.672	0.433	0.494	0.754
**44**	0.231	0.351	0.400	0.220	0.161	0.037	0.968	0.154	0.047	0.278	0.127	1.359
**45**	0.673	1.930	1.978	1.623	0.778	1.433	1.852	2.217	0.864	1.408	0.792	1.950
